# The use of antenatal and postnatal care: perspectives and experiences of women and health care providers in rural southern Tanzania

**DOI:** 10.1186/1471-2393-9-10

**Published:** 2009-03-04

**Authors:** Mwifadhi Mrisho, Brigit Obrist, Joanna Armstrong Schellenberg, Rachel A Haws, Adiel K Mushi, Hassan Mshinda, Marcel Tanner, David Schellenberg

**Affiliations:** 1 Ifakara Health Institute [IHI] (formerly Ifakara Health Research and Development Centre), PO Box 78373, Dar es Salaam, Tanzania; 2 National Institute for Medical Research, Amani Centre, PO Box 81, Muheza, Tanzania; 3 London School of Hygiene and Tropical Medicine, London, UK; 4 Swiss Tropical Institute, Basel, Switzerland; 5 Johns Hopkins Bloomberg School of Public Health, Baltimore, USA

## Abstract

**Background:**

Although antenatal care coverage in Tanzania is high, worrying gaps exist in terms of its quality and ability to prevent, diagnose or treat complications. Moreover, much less is known about the utilisation of postnatal care, by which we mean the care of mother and baby that begins one hour after the delivery until six weeks after childbirth. We describe the perspectives and experiences of women and health care providers on the use of antenatal and postnatal services.

**Methods:**

From March 2007 to January 2008, we conducted in-depth interviews with health care providers and village based informants in 8 villages of Lindi Rural and Tandahimba districts in southern Tanzania. Eight focus group discussions were also conducted with women who had babies younger than one year and pregnant women. The discussion guide included information about timing of antenatal and postnatal services, perceptions of the rationale and importance of antenatal and postnatal care, barriers to utilisation and suggestions for improvement.

**Results:**

Women were generally positive about both antenatal and postnatal care. Among common reasons mentioned for late initiation of antenatal care was to avoid having to make several visits to the clinic. Other concerns included fear of encountering wild animals on the way to the clinic as well as lack of money. Fear of caesarean section was reported as a factor hindering intrapartum care-seeking from hospitals. Despite the perceived benefits of postnatal care for children, there was a total lack of postnatal care for the mothers. Shortages of staff, equipment and supplies were common complaints in the community.

**Conclusion:**

Efforts to improve antenatal and postnatal care should focus on addressing geographical and economic access while striving to make services more culturally sensitive. Antenatal and postnatal care can offer important opportunities for linking the health system and the community by encouraging women to deliver with a skilled attendant. Addressing staff shortages through expanding training opportunities and incentives to health care providers and developing postnatal care guidelines are key steps to improve maternal and newborn health.

## Background

Improving maternal and newborn health requires strengthening of existing evidence-based interventions in antenatal care (ANC) and postnatal care (PNC) (Table 1). Specifically, this includes packages in tetanus immunization, syphilis screening and treatment, and malaria prophylaxis. In developed countries, 97% of women make at least one antenatal visit; 99% deliver with a skilled attendant; and 90% make at least one postnatal visit [1]. In developing countries coverage of at least one ANC visit is relatively high at 69% in Sub-Saharan Africa, compared to 54% in Asia [2]. According to Demographic and Health Survey (DHS) data from 23 African countries, two-thirds of women in Sub-Saharan Africa give birth at home, but only 13% of all women receive a postnatal visit within two days [3]. Although attendance at ANC is encouraging, worrying gaps exist in provision, and coverage statistics are usually based on women who have only one ANC visit, whereas four visits are recommended, and ANC quality varies ([4-6]). Much less is known about the utilisation of PNC, the importance of which has recently been emphasized [4].

**Table 1:** Components of antenatal and postnatal care

**Routine Antenatal Care**
- Focused ANC Visits and referral: 1st visit: before 16 weeks of gestation, 2nd visit: from 20 to 24 weeks of gestation, 3rd visit: from 28 to 32 weeks of gestation & 4th visit: from 36 to 40 weeks of gestation, referral and follow-up should be given to pregnant women with complications.

- Early detection and diagnosis of disease/abnormality ie quick check, history taking, physical examination, laboratory investigation & decision making.

- At least 2 doses of tetanus toxoid vaccination

-Screening and management of pre-eclampsia

- Counseling on health promotion: Intermittent preventive treatment for malaria in pregnancy, insecticide-treated nets, personal hygiene, diet and nutrition, danger signs

- Prevention of mother-to-child transmission (PMTCT) of Human Immunodeficiency Virus (HIV)

- Birth and emergency preparedness: Identify place of birth, preparing essential items, identify at least two blood donors, prepare fund for transport, identify decision maker family members

**Routine postnatal care**
- For the mother: Promotion of healthy behaviours, danger sign recognition and family planning

- For the baby: Promotion of healthy behaviours – hygiene, warmth, breastfeeding, danger sign recognition and provision of eye prophylaxis and immunisations according to local policy

- Extra care for low birthweight babies or babies born to HIV-positive mothers and babies with other special needs.

Adapted from Lawn, J., Kerber, K., 2006. Opportunity for Africa's Newborns: Practical data, policy and programmatic support for newborn care in Africa. eds. PMNCH. Cape Town and Ministry of health-Tanzania: Focused antenatal care malaria and syphilis during pregnancy: Orientation package for service providers. *Ministry of Health and Social Welfare, RCH Unit and NMCP, Dar es Salaam, United Republic of Tanzania; 2004*.

Most maternal deaths occur during labour, delivery or the first 24 hours postpartum, and most intrapartum complications cannot be reliably predicted or prevented, though most can be successfully treated with prompt and appropriate diagnosis and care ([7,8]). The neonatal period is only 28 days but accounts for 38% of all deaths in children younger than 5 years [9]. ANC and PNC have the potential to contribute to reducing maternal and child morbidity and mortality ([10-13]). The World Health Organisation (WHO) has been strongly advocating improvements of maternal health services as part of its Safe Motherhood Initiative (SMI). Regular antenatal care has long been viewed as important for identifying a small minority of women who are at increased risk of adverse pregnancy outcomes and for establishing good relations between the women and their health care providers [14]. The study conducted in India reported that women who had received a high level of antenatal care were about four times as likely to use skilled assistance at delivery compared to women who received low levels of antenatal care [15].

However, poor quality of routine ANC has been documented in terms of its ability to prevent, diagnose or treat complications [10]. Recent studies have challenged the potential of ANC to reduce maternal mortality ([7,16]). Both quality and coverage are essential to maximise impact. Impediments to the effective delivery of ANC and PNC include geographical, financial and cultural barriers ([17-20]). An estimated seven out of every ten women who do not give birth in a facility are not currently receiving PNC [4]. Policies and programs have largely overlooked this critical period, hindering efforts to meet the Millennium Development Goals (MDGs) for maternal and child survival [21].

In Tanzania, where this study was conducted, 94% of women make at least one antenatal visit, but only 47% give birth with a skilled attendant [22] Since 2002 the Tanzania Ministry of Health and Social Welfare (TMoHSW) has promoted the four-visit focused ANC approach ([23,24]). Although pregnant women are advised to start attending ANC before the 16^th^ week of gestation, and services are free, more than 80% of pregnant women initiate ANC later than 17 weeks of gestation ([22,23]).

The postnatal period (or called postpartum, if in reference to the mother only) is defined by the WHO as the period beginning one hour after the delivery of the placenta and continuing until six weeks (42 days) after the birth of an infant [25]. Care during this period is critical for the health and survival of both the mother and the newborn [3]. The 2004–5 Tanzania Demographic and Heath Survey (TDHS) data reports that only 13% of women have the recommended one or more postpartum care visit within two days of delivery, with rates as low as 2% in some regions[22]. Currently, there are no guidelines for postnatal care. The Reproductive and Child Health Section (RCHS) of the Ministry of Health and Social Welfare [Tanzania] is in the process of developing new PNC guidelines to be used country-wide [personal communication with Dr Georgina Msemo, the focal point for neonates, 5^th^ May 2008].

ANC and PNC services are key health interventions for reducing maternal and newborn morbidity and mortality. Although the current rate of ANC uptake is encouraging, detailed information about the actual quality and effectiveness of ANC in practice is scant ([10,26]). This is largely because the packages vary so much from place to place in terms of components, timing, frequency of visits, and provider [27]. Similarly, little evidence is available for the packaging of interventions for routine PNC for mother and newborn ([4,27,28]). Improvement in ANC and PNC can potentially reduce maternal mortality ratio (currently 578 per 100,000 live births) for Tanzania [3] and newborn mortality rate, which is 43 per 1000 live births in the study area ([22,29]). Here we describe the perspectives and experiences of women and health care providers with regard to use of ANC and PNC in order to identify opportunities for improving maternal and newborn health services.

## Methodology

### Study area

The study was conducted in Lindi Rural and Tandahimba Districts in southern Tanzania, a study area that has been described in detail elsewhere ([30,31]). In brief, these areas have a total population of about 300,000 people [32]. Lindi Rural has highland areas as well as low-lying plains with major permanent rivers (Lukuledi, Matandu and Mavuji). There are two main rainy seasons, November to December and February to May. The area has a wide mix of ethnic groups, most common being Yao, Makonde, Mwera and Matumbi. These groups frequently intermarry and are predominantly Muslim. Health services are delivered by the public health system. These consist of a network of dispensaries, health centers and hospitals that offer varying quality of care. There are also a few private not-for-profit dispensaries and hospitals run by Christian mission organisations. Three-quarters of the population live within about 5 km of their nearest facility [29]. Routine immunisation is the basis of the EPI activities. On a regular basis vaccines for measles, diphtheria, pertussis, tetanus, polio and tuberculosis, are provided in health facilities all over the country. Vaccinations are given in static, out-reach, and mobile health facilities. The immunisation schedule including the above vaccines stretches over the child's first year and tetanus vaccination is given to women of childbearing age [33]. In Lindi and Mtwara regions, the proportion of heads of household and women of reproductive age (15–49 years) with no education was 35% and 27% respectively. Thirty-eight percent of a representative sample of 19,007 women aged 15–49 years interviewed in July and August 2004 had experienced the loss of at least one child [29].

### Methods

Data was collected within a framework of ethnographic fieldwork for a larger project assessing community acceptability of intermittent preventive treatment for malaria in infants during March and April 2007. Follow-up data collection was carried out during January 2008. Using a network of female village based informants (VBI) in 8 villages of Lindi Rural and Tandahimba districts ([30,31]), we conducted a series of in-depth interviews (N = 16; N = 8 with VBI, N = 8 with health care providers (HCP)) and focus group discussions (FGD; N = 8).

Each FGD was conducted in groups of 6 to 8 women with babies aged less than one year of age as well as pregnant women with similar backgrounds and experiences [34]. In total, 74 respondents participated in FGD and in-depth interviews. Participants in FGDs included 58 women of whom, 39 had young child less than one year old and 19 were pregnant. Almost all women who participated in FGD and in-depth interviews were aged between 15–42 years and had completed primary school education. Both in-depth interviews and FGD were intended to gather information about the timing and perceived reasons for ANC and PNC; services available in ANC and PNC; perceptions about the importance of ANC and PNC; home births and barriers to ANC and PNC; and lastly, suggestions on how to improve ANC and PNC (see Table 2). The FGD generally took place at the VBI's home. Before the FGD, the moderator introduced all participants, explained the general topics of discussion and encouraged all participants to contribute their ideas. An experienced moderator led the discussions with support from a note-taker, with both taking notes. The FGDs were recorded using an MP3 voice recorder. After the FGD, the note-taker and the moderator reviewed their handwritten notes. After revision of notes, the transcripts were typed and exported to NVivo 2 [35] qualitative data analysis software. Data analysis compared responses from both the in-depth interviews and FGDs. We triangulated responses from in-depth interviews with VBIs and HCPs as well as FGDs with mothers of infants and pregnant women. We found that the responses were in accordance with each other for most of the results. The only exception to this was for barriers to births and suggestions for improvement of ANC and PNC services. For these results we have shown the differences among the sources. Our major key themes emerged as a result of the interview guide (shown in Table 2 below) and the coded transcripts from the FGDs and in-depth interview.

**Table 2:** Questions included in the topic guide used during FGDs and in-depth interviews

**ANC**
1. How early do women go for ANC? Why do they go at that time? (In what month of pregnancy? Why earlier or later?)
2. How often do they go to ANC? Why do they go at that time?
3. What kinds of services do they receive in ANC?
4. Do women think ANC helps their pregnancy?
5. How important do women seem to think ANC is?
6. If women do not go for ANC, what are their reasons?
7. What are the merits and demerits of ANC? What are barriers to accessing ANC?
8. Why do women go to the clinic for ANC, yet mostly deliver at home?
9. Are all mothers abiding to their clinic dates?
10. What in your opinion should be improved?

**PNC**
1. How early do women go for PNC? (In what day/month after delivery?)
2. How often do they go for PNC?
3. What kinds of services do they receive in PNC? Are they satisfied?
4. What do women think PNC does to help their babies and themselves?
5. How important do women seem to think PNC is?
6. If women don't go for post-natal care, what are their reasons?
7. What are the merits and demerits of PNC; what are barriers to accessing PNC?
8. What in your opinion should be improved?

We obtained informed consent verbally at the start of each interview or FGD. Most health care providers were not willing to be recorded, but gave their consent to be interviewed. Interviews with health care providers were done at their workplace and at a time that was convenient for them, particularly when there were few or no clients. In these cases, the analysis was done from written notes. Confidentiality of all study participants was assured and village names have been encoded in this manuscript. We chose a qualitative approach in order to improve our understanding of community views and perceptions regarding ANC and PNC services.

### Ethical approval

The study was undertaken within the framework of the assessment of the community effectiveness of Intermittent Preventive Treatment for malaria in infants (IPTi). We received ethical approval from the local and national institutional review boards (Ifakara Health Institute and the National Tanzania Medical Research Co-coordinating Committee) through the Tanzania Commission for Science and Technology. In addition ethical and research clearance was also obtained from institutional review board of the London School of Hygiene and Tropical Medicine, UK, and Ethics Commission of the Cantons of Basel-Stadt and Basel-Land, Switzerland.

## Results

From the analysis, five major themes emerged (i) Timing and reasons for attending ANC and PNC; (ii) Perceived services available at ANC and PNC; (iii) Perceptions about the importance of ANC and PNC; (iv) Home births and barriers to ANC and PNC; and lastly, (v) Suggestions to improve ANC and PNC. Each theme will be examined separately below.

### Timing and reasons for attending ANC and PNC

Although women are advised to initiate ANC by 16 weeks of pregnancy, and some women had initiated ANC early, the majority began to attend the clinic at or after 17 weeks. Some women were reported to initiate ANC at the 18th or 19th week of pregnancy. Reasons women mentioned for initiating ANC either early or late are shown in Table 3.

**Table 3:** Perceived reasons for ANC by time of attendance

**Perceived reasons for early antenatal care**	**Perceived reasons for late antenatal care**
To confirm early pregnancy	To avoid coming to the clinic many times
To prevent miscarriage	Lack of money
To diagnose and treat illness associated with early pregnancy	Fear of encountering wild animals on the way to the clinic that could cause physical or spiritual harm
To check or monitor the development of the baby	Unsure of being pregnant
To get a clinic card in order to guarantee being attended in case of emergencies	Apathy or laziness *(Uzembe)*
To get advice from the nurses	Being away from one's village or living in a remote place
To avoid being reprimanded by clinic staff for late initiation of ANC	Shyness or embarrassment, particularly among older women and school-age girls

Participants in FGDs and in-depth interviews who understood the importance of early ANC often cited recommendations that care start early:

*"I started going to the clinic when I was three months pregnant. They [health workers] do not allow you to start ANC when you are 6 or 7 months pregnant. They recommend going in the early stages of pregnancy. Up to this moment, I have gone three times" *(FGD, woman, in 9^th^ month of pregnancy, Village T).

HCP's views were in accordance with the women:

*"Women normally attend the ANC four times, I instruct them and educate them on that. The Ministry [of health] has recommended that way so as to avoid problems [in pregnancy]. If they come this month, they do not come next month. This recommendation is now different as women were previously supposed to come every month" *(In-depth interview, HCP Village K).

Despite the widespread availability of free ANC services, most women attend their first antenatal clinic late (after 16 weeks). One of the reasons for a late first visit is to avoid coming many times; that is, if a woman starts early, she will have to attend the clinic many times. Lack of money to start or continue ANC clinic visits was also mentioned by most participants. Women are charged a small amount of money (between 20/- to 50/- Tanzanian Shillings) generally agreed by the community members to support health workers to bring EPI outreach services closer to the women in a specified period. Money was also needed to pay for transport especially for the places where the health facility was a lengthy walk from a woman's village. Other concerns included fear of encountering wild animals on trips to the dispensary where ANC care is provided, and being unsure of pregnancy.

*"I started going to the clinic when I was 5 months pregnant; I was not sure that I was pregnant and therefore decided to go and confirm it" *(FGD, mother with a 1-month-old baby girl, Village R).

*"We tell them to come to the clinic when they are three months pregnant. They normally come three times. For those who live far away, they say they are afraid of wild animals and sometimes they are blocked [from coming] by flooding caused by rains" *(HCP, Village R).

Although in nearly all FGDs and in-depth interviews, respondents reported to have attended ANC services, the trend was not the same for PNC. The majority of those who gave birth at home did seek PNC services, but they reported attending three to seven days after childbirth. However, in several FGD sessions, women with babies as old as two or three weeks had not yet taken their baby for PNC. Their reasons for the delay were mainly due to waiting for the baby's cord stump to fall off, to allow the mother and baby to regain energy lost during childbirth, lack of money and distance to the health facility. The period before the umbilical cord stump falls off is understood to be a period when the baby is particularly vulnerable to harm by jealous or malevolent people and spirits, and the baby is usually secluded inside [31]. PNC was usually perceived as a service for children of all ages, lasting well beyond 42 days after delivery. Respondents in the communities did not make a distinction between the care in the first six weeks and the Expanded Programme on Immunisation (EPI) which is one component of PNC. This confusion may exist because EPI immunizations begin shortly after birth and are first administered during the PNC period. Most respondents viewed postnatal care as very important, justifying taking the child out of the home even during the seclusion period; some respondents reported that a newborn would be taken to the heath facility by relatives, and the mother would be left at home to regain her energy lost during childbirth. Even those women who give birth at health facilities are discharged very soon after birth. A baby is usually taken for follow-up well-baby care services provided through EPI once per month after the first visit.

Respondents had a variety of comments regarding attendance at PNC visits; with HCPs and women being broadly in accordance with one another:

*"There are women who give birth today and decide to take their babies to the health facility the day after. There are also those who give birth on the way to the health facility and decide to take the baby directly to the health facility. However, the majority are those who give birth at home and wait until the cord stump falls off to take their children for PNC services" *(In-depth interview, VBI Village R).

*"There are those who take three to four days and others take up to one week to take their newborn for their first PNC visit. Other women say that they don't have enough energy to go for PNC while some mentioned other physical barriers as an obstacle. Those who live close to the health facility do come within one week while those who live far away may take up to two to three weeks" *(In-depth interview, HCP, Village M)

### Perceived services available at ANC and PNC

During the antenatal period, the most common services women perceived to be important and reported were routinely provided included: weight measurement, physical examination, provision of sulphadoxine-pyrimethamine (SP) for malaria, injection (presumably tetanus toxoid immunization), blood test for syphilis, counseling for birth preparedness, provision of discount vouchers for government-subsidised insecticide-treated nets to prevent malaria, mebendazole tablets for maternal deworming, and iron-folate supplements to prevent anemia. Despite health education in most health facilities, some of these services are poorly understood by pregnant women. The most problematic area was around medication as reflected in some common statements by mothers:

*"I was given three big white tablets, and told to swallow on the spot. I was also given red tablets and a discount voucher, but I was not asked about birth preparedness" *(FGD, woman in 8^th^ month of pregnancy, Village H).

*"We got drugs to prevent or treat anemia and we were examined and provided with a new clinic attendance card *(FGD, mother with 9^th^-month-old baby girl, Village N).

*"I started going to the clinic when I was five months pregnant. I went earlier (2 months) but they [health workers] said that they saw no pregnancy" *(FGD, mother with 9 month-old baby girl, Village N).

The HCP gave a specific list of the services provided at the health facilities, which were in accordance with the mother's perception:

*"They [pregnant women] get tetanus injection, doses of SP (given at two visits) to prevent malaria; the VDRL test for syphilis, an HIV test, urine test, and they also get a discount voucher [for a bed net]" *(In-depth-interview, HCP, Village K).

Postnatal services are perceived to be both important and routinely provided. However, unless there is a serious issue related to maternal complications, these services target the child, and little attention is paid to the mother. The most common services include: weight monitoring, tuberculosis immunisation (BCG), polio vaccination, DPT-1, 2 and 3; and measles vaccination. PNC is perceived by both mothers and HCPs as important for the baby.

*" [...] PNC is just for the child. There is nothing for the mother. All other services that follow soon after birth are for the child" *(In-depth interview, VBI, Village T).

*"My last child was injected and given oral [polio] vaccine, then I was provided with a card. He was also weighed after two weeks. I was asked to come back after a month" *(FGD, woman, in 9^th^ month of pregnancy, Village M).

*"A newborn gets BCG and polio zero soon after birth (majority after day 7) and also gets DPT one and polio one (at day 28) and weight measurement every month. After that a child continues being weighed once every month until he/she gets the measles vaccination and a discount voucher" *(In-depth-interview, HCP, Village N).

### Perceptions about the importance of ANC and PNC

The majority of the women interviewed attended ANC. Nearly all women perceive ANC services to be important and expressed complete trust in HCPs and the care they receive. However, a few women do not understand the importance of care provided. The most commonly mentioned assistance given to pregnant women included advice about care of their pregnancies; assessment of fetal vital status; ascertainment of fetal position; maternal vaccination; provision of vouchers for bed nets to prevent malaria; blood tests to diagnose disease and assess health status. As a routine part of ANC, women receive a clinic card which is crucial in case of an unforeseen complication that requires hospital attendance. The following excerpts describe HCPs and women's varied perceptions of the benefits of ANC:

*"ANC services are quite helpful. For example, when I was pregnant my baby was lying in the wrong position and they helped her turn for a safe delivery" *(FGD, mother with a 9^th^- month-old baby girl, Village K).

*"I haven't known any pregnant woman who is so careless as to miss ANC. But carelessness about seeking postnatal care is common, for example, not taking a child for PNC services" *(FGD, woman, in 8^th^ month of pregnancy, Village H).

*"I am just afraid of being denied services when I need them, so one must just go [to ANC] to get the [clinic] card. If you do not have a card, they will not accept you when there is a problem.... Otherwise, we could just rest at home" *(FGD, woman, in 9^th^ month of pregnancy, Village C).

*"They [women] are happy about the services provided and they perceive them to be helpful. Those who get referral do say thanks to us for the prior advice given although there are some who still give birth at home" *(In-depth-interview, HCP, Village M).

In most in-depth interviews and FGDs, women reported that PNC services were good for the child, and that this is why most children are taken for PNC. Women perceived these services to be effective: in almost all FGDs and in-depth interviews, women reported that PNC prevents children from getting fever, tuberculosis, measles, malaria, polio, whooping cough, tetanus and diphtheria. In addition, respondents reported that weight monitoring helps to understand if the child is growing in the right way. In some FGDs respondents said they believed that HCPs understand the benefits of PNC services, which is why mothers take children to HCPs for PNC. A few women reported not taking their children to the health facility for PNC because of a belief that an injection could harm their child, but these views seemed the exception rather than the rule. The following excerpts describe the benefits women perceive PNC has for their children and their trust in HCPs:

*"I go for postnatal care so that I can monitor the weight of my child. This helps us to feed the baby properly if her weight decreases" *(FGD, mother with 2-week-old baby girl, Village N).

*"These services [PNC] are helpful but we do not understand exactly what they help. It is the healthcare provider who knows." *(FGD, woman in 8^th^ month of pregnancy, Village T).

In a few cases, HCPs views contrasted with those of the women:

*"Some women can just refuse that their children be injected. For example, the people who live in remote areas do not want their children to be injected because they believe injections can cause convulsions. We have to educate them through health education sessions" *(In-depth interview, HCP, Village R).

### Home births and barriers to ANC and PNC services

Although the vast majority of women attend ANC services, more than half give birth at home. The major barriers reported for home as opposed to facility-based birth include lack of money, distance to the health facility, fear of caesarean section at the health facility and lack of privacy or a dedicated labor room at the health facility. Giving birth at the hospital is perceived by the women to be associated with severe delivery complications. However, the HCP had differing views on the reasons for giving birth at the health facility:

*"There are women who are frightened to go to the hospital when they are referred because they want to avoid a *caesarean section: *referral means a *caesarean section *at the hospital. The prolonged labour in our district hospital can lead to *caesarean section. *Formerly, I gave birth at home because other women in my neighborhood warned me this could happen [if I went to the hospital]" *(FGD, woman in 9^th^ month of pregnancy, Village T).

*"There are women who think that when they are referred to hospital, it means they will need an operation *[caesarean section]. *It is believed that if a woman experience prolonged labour at the hospital, c-section can take place immediately. Most women are not happy because some of them have to work and maintain their household; who will do their work [while they recover]? If someone has surgical delivery then she must rest for about six to eight months" *(In-depth interview, VBI, village R).

*"Although I usually advise women who come here [to the clinic] to give birth at the nearest health facility they still give birth at home because we have not moved to our new building. But when things go wrong at home, only then do they come to see us. We have an unfinished building, and there is no special bed for delivery and no delivery kits" *(In-depth interview, HCP, Village K).

Although there is a delay in starting PNC, nearly all women eventually attend the clinic for their child to receive services. Most mothers concur about the importance of PNC and encourage each other to attend the clinic. Most women said that they had not heard of anyone whose baby had not been taken for PNC. However, long distances and inaccessibility to a health facility (especially during the rainy season), negligence and unplanned pregnancy were important barriers to use of PNC. Some people who live on farms were reported to go to the health facility solely to get a clinic card to facilitate later care in case emergency care was needed at a later date. It was also reported that children are taken for PNC up to the age of one year. The HCP's views were in accordance with the women's perceptions on PNC services:

*"I haven't heard of anyone missing postnatal clinic. But those who live in the farmland, they just go to show the baby so that they can get a clinic card [in case care is needed later]" *(FGD, mother with a 3 month-old baby boy, Village H).

*"Other women do not take their children for postnatal care. A child is taken for postnatal care up to the age of one year. After this, the child is seen as too old to be taken for postnatal care" *(FGD, woman, in 8^th^-month of pregnancy, Village T).

*"Some women who live far away from the clinic are less likely to attend PNC, especially during the rainy season. Usually those who live in the village attend; they are not lazy at all. When they arrive I usually ask them about their reasons for not attending PNC on time" *(In-depth interview, HCP, Village K).

### Suggestions for improvement

#### Perspectives of mothers

Different suggestions were mentioned in the FGDs and in-depth interviews to improve ANC and PNC. Staff shortages, lack of equipment and supplies and wastage of time at the clinic were the complaints that dominated conversations in nearly all FGD and in-depth interviews. Most rural health facilities have a shortage of skilled health providers. Women perceived that because of this staff shortage, they spend a lot of time during each ANC or PNC visit. In addition, respondents suggested furnishing all health facilities with adequate and appropriate supplies and equipment, such as scales for weighing pregnant women and babies. Moreover, women suggested that health care providers should use more polite language while interacting with their clients, as verbal abuse and condescension were common complaints.

*"Those who go for weight monitoring spend less time at the clinic than those who go for vaccination. This is because there is one health care provider; we suggest that there is a need to increase the number of health care providers" *(FGD, mother with one-year-old baby boy, Village K).

*"When we go late to the clinic for PNC, the health care providers complain that they are getting a meagre salary and yet we keep bothering them" *(FGD, mother with 9^th^ -month-old baby boy, Village N).

*"We request that health care providers behave more kindly so as to respond positively and politely to their clients" *(FGD, woman, in 9^th^-month of pregnancy, Village T).

#### Perspectives of health care providers

In nearly all in-depth interviews with health care providers, respondents suggested that incentives such as refresher courses should be offered to improve job skills. They also complained about their workload, inadequate equipment and poor remuneration.

*"We have no essential equipments such as a weighing scale or labour kits for childbirth. We have stopped providing DPT- Hepatitis B vaccine because we have no syringes"*. (In-depth interview, HCP, Village H).

*"We have been trained to offer rapid plasma regain (RPR) but this service is not offered in this dispensary. We need radio call to communicate easily; there is a lot of work so we need at least two nurse midwifes and our salary is also not enough" *(In-depth interview, HCP, Village M).

The majority of the respondents also suggested that all ANC services be provided in all health facilities: they complained that services such as Prevention of Mother-To-Child Transmission (PMTCT) for HIV infection and RPR test for syphilis are not available in all health facilities.

## Discussion

We found that many women had a positive attitude towards antenatal and postnatal care. However despite the perceived benefits of postnatal care, there was a total lack of postnatal care for the mothers. Women were not able to differentiate between the care in the first six weeks and the Expanded Programme on Immunisation which continues for the first year or more of a child's life. Both mothers and HCPs mentioned shortages of staff, equipment and supplies at clinics as a major priority to improve ANC and PNC. Both mothers and health care providers recommended that all antenatal services be provided at all levels of care.

The study was based on a small and purposive sample and as a result may not be representative of the entire population seeking ANC and PNC in rural Southern Tanzania. Still, this study provides important preliminary insights into many factors that shape community acceptance and utilisation of antenatal and postnatal care services. Moreover, the use of qualitative techniques enabled us to gain a better understanding of community views and perceptions regarding ANC and PNC services than was previously available. By examining information collected from FGDs and in-depth interviews with mothers and health care providers, we were able to increase the validity of our study. This study identified important research gaps including the need to measure the prevalence of negative practices, such as women being turned away by the health care providers for initiating ANC too early or late. There is a need to explore strategies for providing postnatal care for mothers and newborns, particularly to reach mothers and their babies after home birth. There is also an urgent need to explore community-based financing schemes to help alleviate transport problems for women experiencing obstetric emergencies or for sick newborns.

As central strategies of the Safe Motherhood Initiative, ANC and PNC have the potential to improve maternal and child health, including neonatal care practices in the home ([36,37]). Women in these communities are receptive to information about pregnancy and infant care through varied communication channels during the preconception, antenatal and postnatal periods ([38,39]). Several barriers such as lack of money, distance to the health facility, lack of privacy to attending ANC and PNC that were mentioned are in line with the findings from other studies ([6,18-20,40,30,43]). Moreover, it was also mentioned that fear of encountering wild animals on the way to the clinic was reported as a factor hindering intrapartum care-seeking from health facilities. This may imply that health services should be brought close to the people. Although knowledge regarding the importance of ANC and PNC was high, providing adequate healthcare alone does not guarantee the improvement of women's health [44]. Attention must be paid to the social and economic conditions that keep women from exercising their right to utilise existing services [45]. When women are knowledgeable about different modes of treatment they are more inclined to insist upon their rights and demand choices [46].

Although women are advised to initiate ANC early, the majority started to attend after the 16^th^ week of pregnancy. The whole issue of reporting gestation period in weeks poses a methodological challenge. Normal women report their gestation period in months as it is easier for them to recall and not in weeks. In this study we used months rather than weeks. Late attendance, four months and above, did sometimes lead to being chastised by the health care providers or being denied ANC altogether for starting ANC too late. Our results support the findings of others ([10,20,42]) which point out that the majority of the interviewed women attend ANC to get antenatal attendance cards to facilitate prompt care in case of complications later in the pregnancy. Early ANC can, however, be used effectively to monitor the progress of the pregnancy. This can be done early through establishment of woman's baseline data such as blood pressure, syphilis/HIV screening and body mass index to detect and treat adverse pregnancy related outcomes [47]. Early booking can also help to provide other pregnancy-related services such as nutritional education, tetanus immunization, iron and folic acid tablets, insecticide-treated nets and malaria prophylaxis on time. Low frequency of visits or late timing of the first antenatal visit are undesirable because they limit the amount and quality of care that a pregnant woman receives [18]. A study conducted in Mexico City found that an inadequate number of visits was associated with 63% higher risk of intrauterine growth retardation [48]. Frequent antenatal care attendance would have an impact on maternal mortality not only through early detection of obstetric conditions but also by influencing women's decision to deliver babies at health facilities [49].

PNC coverage is limited by the cultural tradition of keeping the baby indoors, especially among women who gave birth at home. This tradition of seclusion has also been reported elsewhere, ([3,17,31]). Policymakers should therefore consider delivering PNC at both health facilities and at home to overcome financial, geographical and cultural barriers to care-seeking outside the home during the early postnatal period ([17,50]). Although PNC was reported to be limited to neonates and infants, the reality is that there was no effective PNC, even for the newborns. The confusion about the duration and components of PNC among women in the study population with well-baby care was mainly due to the lack of comprehensive PNC services and could undermine prompt care-seeking for mother and the baby. Efforts are needed to speed up development of a new PNC guideline to help educate mothers that they are also vulnerable to infection during the immediate postpartum period and would benefit from postpartum care seeking ([25,51]).

During the antenatal period, certain services such as routine weighing and vaccination are perceived to be important. Despite availability of information about the component services of ANC, some drugs and vaccinations provided during clinic visits are poorly understood by pregnant mothers, suggesting that health care providers' knowledge and strategies for detecting early pregnancy and informing their clients about these drugs and services need to be improved. Although there were a few reports of women not taking their children to the health facility for PNC, most women trust that health care providers understand the purpose and benefits of the services, they are providing, a finding supported by other studies [52]. Through sharing of knowledge with women and mothers in health education session during ANC and PNC visits [53], health care providers can influence health-seeking behaviour of pregnant women in this rural setting. However, poorly motivated health workers seem unlikely to care too much about the quality of service they are providing [54]. Recent studies document that behaviour change communications during ANC can work to promote evidence-based neonatal care practices, care-seeking and demand for skilled intrapartum and postnatal care, particularly in developing countries ([55,56]).

Potential opportunities exist to improve ANC and PNC services as suggested by both health care providers and clients. Client awareness of, demand for, and perceived benefits of ANC and PNC are high, but the quality of service needs to be strengthened. This will entail improving access to services; providing training opportunities for health care providers as well as behavior change communication strategies to overcome cultural barriers. Health workers' negative attitudes have frequently been a complaint and a reason cited for lower utilization of health services ([57-59,30]). Poor treatment, or poor quality of care during clinic visits, discourages care-seeking strategies and erodes trust in health care providers [6]. Approaches to improving quality of care should be based on regular quality assessments and additional operational research activities [60].

To increase the availability and effectiveness of ANC and PNC services, staff shortages and skills gaps, lack of equipment and supplies, and the absence of proper PNC guidelines must be remedied. Suggestions from most respondents focused on increasing the number of staff at all levels of health facilities. The importance of strengthening human resources in healthcare is also increasingly acknowledged ([57,61,62]). Better living conditions for health care providers, as well as incentives for good job performance would improve delivery of health services ([63-65]).

## Conclusion

Despite some gaps in utilization, ANC and PNC are viewed positively. They offer important opportunities to encourage women to deliver with a skilled attendant in a health facility, and function as an entry point for care from birth through childhood and into adulthood. However, a number of findings suggest the need for additional research and program action. Some women reported their only reason for attending ANC was to get an antenatal attendance card, or PNC to get a growth monitoring card, in order to ensure curative health services would be provided in an emergency. Efforts need to be made to communicate the benefits of ANC and PNC more effectively. PNC services for mothers who recently delivered are either widely underutilised or unavailable. Efforts should be made at a programmatic and policy level within the formal health care system to provide this care to women and their newborns. Innovative behavior change and service delivery strategies must be designed and tested to provide postnatal care during the period immediately after birth when newborns are secluded in the home. Shortages of staff, equipment and supplies, difficulty accessing health facilities, and lack of clear guidelines on PNC need to be addressed in order for ANC and PNC to achieve their full potential for maternal and newborn health.

## Abbreviations

ANC: Antenatal Care; BCG: Bacille Calmette-Guerrin; DHS: Demographic and Health Survey; DPT-HB: Diptheria, Pertussis, Tetanus and Hepatitis B vaccine; FGD: Focus Group Discussion; EPI: Expanded Program on Immunisation; HCP: Health Care Provider; HIV: Human Immunodeficiency Virus; IHI: Ifakara Health Institute; IPTi: Intermittent Preventive Treatment for malaria in infants; MDG: Millenium Development Goal; PMTCT: Prevention of Mother to Child Transmission of HIV; PNC: Postnatal Care; RCHS: Reproductive and Child Health Section; RPR: Rapid Plasma Reagin; SP: Sulphadoxine-Pyrimethamine; SMI: Safe Motherhood Initiatives; VBI: Village Based Informant; VDRL: Venereal Disease Research Laboratory; WHO: World Health Organisation.

## Competing interests

The authors declare that they have no competing interests.

## Authors' contributions

MM, BO, JAS, DS, HM and MT devised the study design and objectives. MM, JAS, AKM, HM and DS contributed to data collection, analysis and interpretation. MM did the data collection, analysis and wrote the first draft of the manuscript. BO, RAH and HM provided technical support. All authors read, commented on and approved the final manuscript.

## Pre-publication history

The pre-publication history for this paper can be accessed here:


